# Exosomal Long Non-Coding RNA: Interaction Between Cancer Cells and Non-Cancer Cells

**DOI:** 10.3389/fonc.2020.617837

**Published:** 2021-01-14

**Authors:** Shenqi Han, Yongqiang Qi, Yiming Luo, Xiaoping Chen, Huifang Liang

**Affiliations:** ^1^ Hepatic Surgery Center, Tongji Hospital, Tongji Medical College, Huazhong University of Science and Technology, Wuhan, China; ^2^ Hubei Key Laboratory of Hepato-Pancreato-Biliary Diseases, Wuhan, China; ^3^ Key Laboratory of Organ Transplantation, NHC Key Laboratory of Organ Transplantation, Key Laboratory of Organ Transplantation, Ministry of Education, Chinese Academy of Medical Sciences, Wuhan, China

**Keywords:** extracellular vesicle, exosome, long non-coding RNA, tumor microenvironment, cancer

## Abstract

Exosomes are small membranous vesicles released by many kinds of cells, and are indispensable in cell-to-cell communication by delivering functional biological components both locally and systemically. Long non-coding RNAs (lncRNAs) are long transcripts over 200 nucleotides that exhibit no or limited protein-coding potentials. LncRNAs are dramatic gene expression regulators, and can be selectively sorted into exosomes. Exosomal lncRNAs derived from cancer cells and stromal cells can mediate the generation of pre-metastatic niches (PMNs) and thus promote the progression of cancer. In this review, we summarized the fundamental biology and characteristics of exosomal lncRNAs. Besides, we provided an overview of current research on functions of exosomal lncRNAs between cancer cells and non-cancer cells. A deep understanding of exosomal lncRNAs’ role in cancer will be facilitated to find important implications for cancer development and treatment.

## Introduction

Exosomes are extracellular vesicles (EVs) with a size range of ~40 to 160 nm (average ~100 nm) in diameter from multivesicular bodies (MVBs) fusing with plasma membrane (1). In 1983, Johnstone RM et al. firstly discovered EVs in mature sheep reticulocytes, and named them as exosomes. They considered exosomes as cellular “debris” at that time (2). In 1996, Raposo et al. found that B lymphocytes secreted antigen-presenting exosomes which induced T cell response (3). In 2007, Valadi H. et al. put forward that exosomes containing both messenger RNAs (mRNAs) and microRNAs (miRNAs) can be transferred to another cell, and have function in the new location (4). Subsequently, more and more studies indicated that exosomes can mediate intercellular communication by carrying proteins, DNAs, and RNAs including non-coding RNAs (5). In addition, exosomes were presented in vast majority of body fluids, including plasma (6), urine (7), saliva (8), ascites (9). In tumor milieu, exosomes were derived among different kinds of cells like tumor cells, fibroblasts, and immune cells (10, 11), regulating tumor microenvironment (TME) mainly by autocrine, paracrine, or endocrine way (12). On account of the distinctive role in tumor development and the universality of existence, exosomes high prospects as therapeutic targets as well as the diagnosis biomarkers in cancer.

Long non-coding RNAs (lncRNAs) are defined as transcripts longer than 200 nucleotides that have no or limited protein-coding capacity (13). Owing to highly heterogeneous primary sequence and low expression level, lncRNAs were once believed as transcriptional “noise” (14). Thanks to high-throughput sequencing technology, it is now evident that lncRNAs have formidable functions in regulating gene expression and cell homeostasis. LncRNAs are located in either the cytoplasm or nucleus, which can interact with microRNAs, mRNAs, RNA-binding proteins (RBPs), transcription factors and chromatins, and act as enhancer-like RNAs (15, 16). The complex and extensive roles of lncRNAs in gene regulation are commonly separated into epigenetic, transcriptional and post-transcriptional levels (17). Beyond that, lncRNAs are reported to encode hidden polypeptides by the translation of small open reading frames (smORFs) (18–20). It should be emphasized that lncRNAs function as competing endogenous RNAs (ceRNAs) by sponging microRNAs, and hence inhibit microRNAs interacting with target mRNAs (21, 22). CeRNAs represent a new means of mechanism that involve in two kinds of non-coding RNAs in the same physiological process, which were largely showed in exosomal lncRNA regulation. A significant portion of lncRNAs are oncogenic lncRNAs that are associated with cancer occurrence, progression and outcome. Emerging evidence support the notion that lncRNAs play indispensable characters in proliferation (23), apoptosis (24), metastasis (25), angiogenesis (26), metabolism (27) of cancer (28). For example, lncRNA PTAR upregulated ZEB1 by competitively binding miR-101-3P like sponges, promoting TGF-β induced EMT and invasion in ovarian cancer (29). The diversity of lncRNAs function and mechanism implies a great potential in tumor malignant transformation, and exosomes amplify the function of lncRNAs by means of transporting them to distal region. As a novel way of acting, lncRNAs can be selectively sorted into exosomes and serve as signaling messengers in intercellular communication (4). LncRNAs were wrapped by exosomes and delivered to recipient cells, and then converted cell phenotypes by aforesaid mechanisms. In TME, exosomal lncRNAs have crucial impacts on proliferation, metastasis, angiogenesis, immunosuppression, and chemoresistance. It is fortunate that exosomes’ lipid membranes protect lncRNAs from degradation by ribonuclease. As a result, lncRNAs can be delivered to primary tumor tissue or distant organs safely (30). Therefore, the application of exosome-derived lncRNAs in tumorigenesis, development and treatment has attracted growing attention (31). In this review, we not only summarized basic information of exosomes, but also focused on the latest literatures related to the exosomal lncRNAs in cancers.

## Exosome: Generation and Uptake

Exosomes originate from early and late-sorting endosomes formed by inward budding of the cell membranes (32). Subsequently, Late-sorting endosomes mature into MVBs (33). During this process, the endosomal limiting membranes inwardly invaginate and envelope proteins, RNAs and DNAs to form intraluminal vesicles (ILVs), which are the exosomes released to extracellular space subsequently. According to the condition and environment of the cells, MVBs would secret ILVs as exosomes by fusing with cell membrane, which is regulated by several RAB GTPases (including RAS-related protein RAB5, RAB7, RAB11, RAB27, and RAB35) as well as membrane fusion soluble *N*-ethylmaleimide-sensitive factor attachment protein receptor (SNARE) complex proteins (34). It’s worth mentioning that lncRNAs are involved in the biogenesis of exosomes and tumor development. For example, Wang, F. W. et al. illuminated that lncRNA-APC1 can bind Rab5b mRNA and reduce its stability, leading to reduction of exosomes production, thereby inhibiting colorectal cancer (CRC) growth, metastasis, and angiogenesis (35). Also, MVBs can fuse with lysosomes or autophagosomes to be degraded. The generation of exosomes mainly involves in two sorting mechanisms, including endosomal sorting complex required for transport (ESCRT) pathway and the ESCRT independent pathway (36, 37). However, it is still unclear whether the production of the same exosomes can contain these two sorting methods, or whether the two sorting methods can coexist in one type of cells.

**Figure 1:**
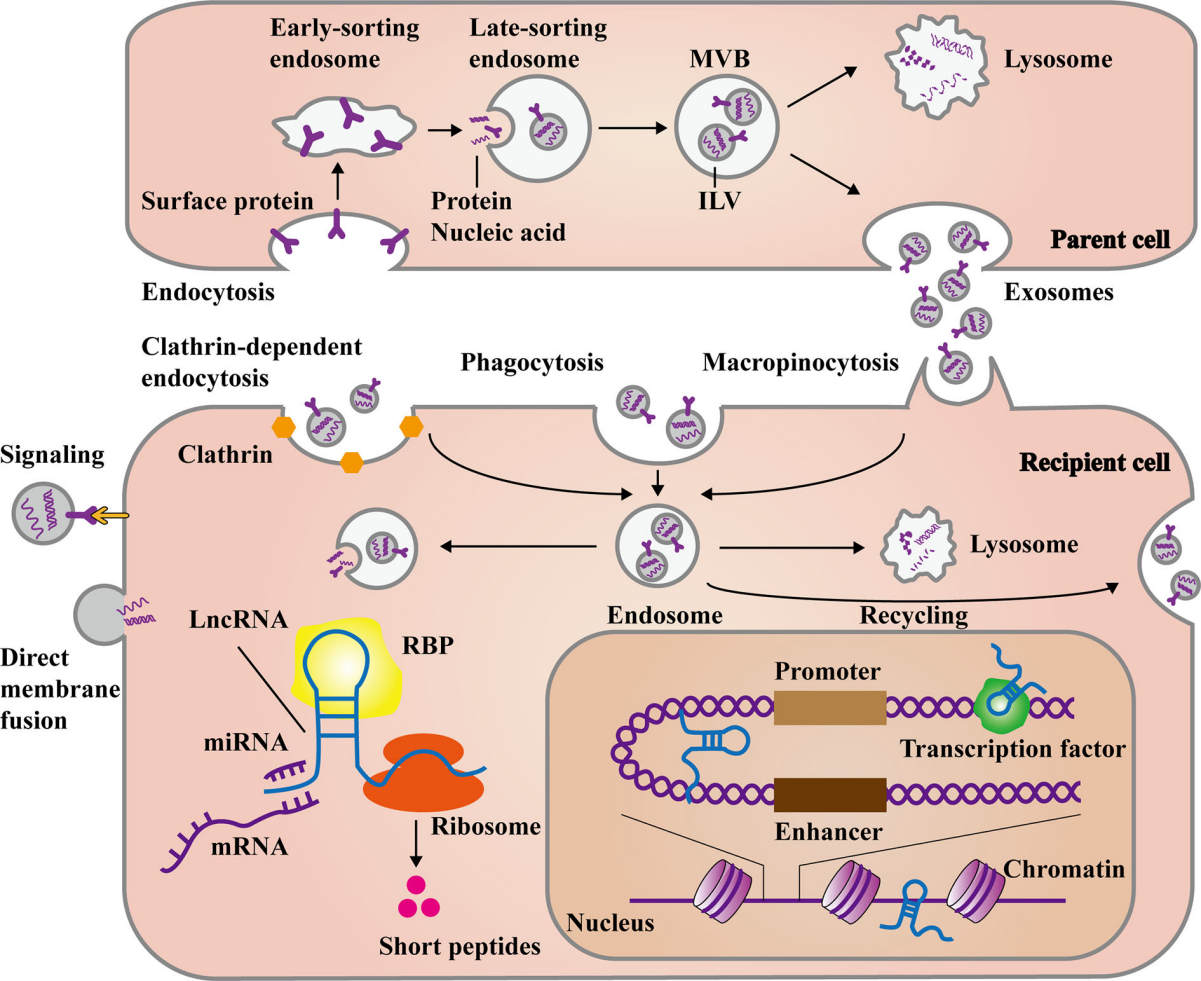
The main process of exosomal long non-coding RNAs (lncRNAs) formation and release. The formation of exosomes stems from the endocytosis of some membrane proteins at the plasma membrane, forming early and late-sorting endosomes. Then intraluminal vesicles (ILVs) are formed through the inward budding of the late-sorting endosomal membrane with encapsulating the substances like proteins, DNAs and RNAs. Finally, late-sorting endosomes mature to multivesicular bodies (MVBs), which release ILVs as exosomes. Otherwise, they fuse with lysosomes to be degraded. Exosomes can directly recognize and transmit signal to recipient cells, as well as fuse with plasma membrane to release cargos. In the other hand, exosomes can be internalized to form endosomes mainly through clathrin-dependent endocytosis, clathrin-independent macropinocytosis, or phagocytosis. Deeping on the needs of cells, the endosomes release exosomal cargos, fuse with lysosomes for degradation, or even fuse with plasma membrane again to recycle exosomes. The exosomal lncRNAs are subsequently released to regulate cell function in various ways. In the cytoplasm, lncRNAs can affect post-transcriptional levels by binding miRNAs, mRNAs, and proteins. Besides, some lncRNAs can even encode short peptides. In the nucleus, lncRNAs can also interact with transcription factors and chromatins, as well as act as enhancer-like RNAs.

The capturing and uptaking of exosomes are firstly divided into two ways according to whether they enter the cell. One is to rely on the interaction of glycans, lectins, integrins, and other cell adhesion molecules on the surface of exosomes with the cell membrane to directly activate the signaling pathway, or have fusion with the cytoplasm membrane and release cargos into the cytosol (38, 39). The other is be internalized by cells through endocytosis to form endosomes (40), which mainly includes clathrin-dependent endocytosis and clathrin-independent macropinocytosis (41) or phagocytosis (42). Endosomes can release exosomes into the cytoplasm and further release the contents including lncRNAs. The released lncRNAs can exert regulatory effect through various mechanisms, which include sponging miRNAs to regulate target genes, participating alternative splicing or RNA editing by matching with mRNAs, and integrating with RNPs. Besides, lncRNAs can even act as protein-coding RNAs to translate short peptides. In the nucleus, lncRNAs can also interact with transcription factors to promote or suppress gene transcription, induce chromatin remodeling and histone modification, as well as function as enhancers (43–45). What’s more, Endosomes can be degraded by fusing with the lysosomes, or even re-fusing with the cell membranes to release exosomes outside the cell again (**Figure 1**). However, it is unknown whether the release of endogenous generated and exogenous captured exosomes occur together or separately. *In vitro* and *in vivo* experiments have shown that exosomes were more likely to be absorbed by parent cells and can be used to make targeted drug carriers (46). Exosomes’ generation and uptake ultimately depend on the cell type and environment. Therefore, an acknowledged mechanism is requisite to help us further understand the role of exosomes in cell-to-cell communication.

## Exosomal Long Non-Coding RNA: Sequencing and Database

The confirmation of exosome derived lncRNA is the first step to start the research, and the ways to obtain the appropriate lncRNA worth studying are various and evolutionary. Four to five years ago, microarray once occupied the mainstream, Qu, L. et al. utilized lncRNA microarray to compare lncRNA expression profiles between parental and sunitinib resistant RCC cells, and finally confirmed the most discrepant lncRNA (47). In recent years, as the cost of high-throughput sequencing decreased, its advantages of high sensitivity, whole-genome coverage, and the ability to explore unknown sequences have been amplified, resulting in a significant increase in high-throughput sequencing applications (48). For example, Yu, S. et al. performed extracellular vesicle long RNA-seq (including lncRNA) on plasma samples collected from 501 subjects, and developed 8 long RNAs for the detection of pancreatic cancer (49). In addition, there are also studies based on star lncRNAs such as HOTAIR and H19 to explore their roles with exosomes in TME (50, 51). Nowadays, a series of exosome databases that collect various public exosome sequencing data are constructed. In exoRBase (http://www.exoRBase.org), 58,330 circular RNAs (circRNAs), 15,501 lncRNAs, and 18,333 mRNAs derived from RNA-seq data analyses of human blood exosomes and experimental validations from published literature are concluded (52). Besides, The exRNA Atlas (http://exrna-atlas.org) is the data repository of the extracellular RNA communication consortium (ERCC), including 7,570 small RNA sequencing and qPCR-derived exRNA profiles from human and mouse biofluids (53). The combination of high-throughput sequencing and exosome databases is conducive to understand the profile of exosomal lncRNAs under specific pathophysiological conditions, facilitating efficient screening of exosomal lncRNAs worthy of study.

## Exosomal Long Non-Coding Rna: Functional Roles Between Cancer And Non-Cancer

Intercellular signaling interaction are constructed between cancer cells and non-cancer cells to accelerate malignant progression of cancer. Furthermore, intracellular signaling networks operate by integrated circuits to reprogram gene expression, which induce hallmark capabilities of cancer, such as sustaining proliferation and activating invasion (54). Originally, cell communicated through direct cell-to-cell contact and soluble factors (55). Now, exosomes emerge as vital participants in the intercellular signaling transmission. LncRNAs are key functional molecules that mediate intercellular signaling interaction due to the role in genetic and epigenetic modulation. Hence, growing enthusiasm and energy are devoted to investigating the specific role and mechanism of exosomal lncRNAs in cancer.

In exosomes associated TME, “seed-and-soil” hypothesis (56) has far-reaching implications. The exosomes secreted by tumor cells carry various inflammatory factors and immunosuppressive factors (“fertilizer”), such as macrophage migration inhibitory factor (MIF) (57) and PD-L1 (58), which perform in the surrounding or distant normal tissues or organs, causing vascular leakiness (59), inflammation infiltration (60), extracellular matrix (ECM) remodeling (61), and immune suppression (62). A series of stimulation transform the non-tumor environments into pro-tumorigenic pre-metastatic niches (PMNs) (“soil”), attracting tumor cells (“seeds”) to colonize and grow. Concretely speaking, activated stromal cells can release a lot of cytokines and chemoattractants through exosomes, such as IL-6, IL-8 (63), and S100A9 (64), which trigger the proliferation, invasion, stemness, and chemoresistance of tumor cells. In addition, among tumor cells of different malignant degree, exosomes will also be delivered by paracrine to enhance the overall metastatic burden. Consequently, we divided exosomal lncRNA associated studies into three categories: cancer to non-cancer, cancer to cancer, and non-cancer to cancer (**Table 1**), which are beneficial to understand the unique and important roles of exosomal lncRNAs in each stage of the interaction between cancer cells and non-cancer cells, so as to provide ideas for the development of targeted diagnostic methods and treatment strategies.

**Table 1:** The biological function and mechanism of exosomal long non-coding RNAs (lncRNAs) in cancer.

LncRNA	Parent cell	Recipient cell	Biological function	Mechanism	Year	Ref
Cancer cell to non-cancer cell						
LncRNA BCRT1	Breast cancer cells	Macrophages	Promote M2 polarization	Increase TGF-β and chemotaxis	2020	(65)
LncRNA SNHG16	Breast cancer cells	γδ1 T cells	Induce CD73+γδ1 T cells and immunosuppression	Activate TGF-β1/SMAD5 pathway by sponging miR-16-5p	2020	(66)
LncRNA LNMAT2	Bladder cancer cells	Endothelial cells	Promote angiogenesis	Induce H3K4me3 on PROX1 promoter by binding hnRNPA2B1	2020	(67)
LncRUNX2-AS1	Multiple myeloma cells	Mesenchymal stem cells	Repress osteogenesis	Decrease RUNX2 by binding pre-mRNA and reducing the splicing efficiency	2018	(68)
LncRNA H19	CD90+ liver cancer cells	Endothelial cells	Promote angiogenesis and cell-to-cell adhesion	Increase VEGF	2015	(69)
LncRNA‑ATB	Glioma cells	Astrocytes	Promote migration and invasion	Bind and suppress miR‑204‑3p	2019	(70)
LncRNA TUC339	Liver cancer cells	Macrophages	Promote M2 polarization	Interact with cytokine-cytokine receptor, bind CXCR chemokine receptor and signal Toll-like receptor	2018	(71)
LncRNA-POU3F3	Glioma cells	Endothelial cells	Promote angiogenesis	Increase bFGF, bFGFR, and angio	2017	(72)
Linc-CCAT2	Glioma cells	Endothelial cells	Promote angiogenesis	Increase VEGFA, TGF-β, and Bcl-2, decrease Bax and caspase-3	2017	(73)
LncRNA HOTAIR	Glioma cells	Endothelial cells	Promote angiogenesis	Increase VEGFA	2017	(51)
LncRNA MALAT1	Epithelial ovarian cancer cells	Endothelial cells	Promote angiogenesis	Increase VEGF-A, VEGF-D, ENA-78, PlGF, IL-8, angiogenin, bFGF, and leptin	2017	(74)
Cancer cell to cancer cell						
LncRNA-ARSR	Sunitinib-resistant RCC cells	Sunitinib-sensitive RCC cells	Transmit sunitinib resistance	Increase AXL and c-Met by sponging miR-34 and miR-449	2016	(47)
LncRNA AFAP1-AS1	Trastuzumab-resistant breast cancer cells	Trastuzumab-sensitive breast cancer cells	Transmit trastuzumab resistance	Interact with AUF1 and increase ERBB2 translation	2020	(75)
LncRNA MRPL23-AS1	High invasive SACC cells	Low invasive SACC cells	Promote EMT, migration, and invasion	Induce H3K27me3 on E-cadherin promoter by binding EZH2	2020	(76)
LncRNA SBF2-AS1	TMZ-resistance GBM cells	TMZ-sensitive GBM cells	Transmit TMZ resistance	Decrease XRCC4 by sponging miR-151a-3p, leading to DSB repair suppressed	2019	(77)
Lnc-Sox2ot	High invasive PDAC cells	Low invasive PDAC cells	Promote EMT and stemness	Increase Sox2 by sponging miR-200 family	2018	(78)
LncRNA-UCA1	Hypoxic bladder cancer cells	Normoxic bladder cancer cells	Induce EMT, promote proliferation, migration, and invasion	Increase vimentin and MMP9, decrease E-cadherin	2017	(79)
Lnc-MMP2-2	TGF-β pretreated lung cancer cells	Normal lung cancer cells	Promote migration and invasion	Increase MMP2	2018	(80)
Linc-ROR	Thyroid cancer stem-like cells	Normal thyroid cancer cells	Induce EMT	None	2017	(81)
LncRNA ZFAS1	High invasive gastric cancer cells	Low invasive gastric cancer cells	Promote proliferation, migration, and EMT	Increase cyclinD1, N-cadherin, slug, twist, p-ERK and Bcl-2, decrease E-cadherin and Bax	2017	(82)
Non-cancer cell to cancer cell						
LncRNA HISLA	TAMs	Breast cancer cells	Promote aerobic glycolysis, apoptotic resistance, and chemoresistance	Block the interaction of PHD2 and HIF-1α, inhibit the hydroxylation and degradation of HIF-1α	2019	(83)
LncRNA H19	CAFs	Colorectal cancer cells	Promote stemness and chemoresistance	Act as a ceRNA for miR-141, activate β-catenin pathway	2018	(84)
LncRNA PTENP1	Normal cells	Bladder cancer cells	Promote apoptosis, inhibit migration and invasion	Decrease PTEN by sponging miR-17	2018	(85)

RCC, renal cell carcinoma; SACC, salivary adenoid cystic carcinoma; TMZ, temozolomide; GBM, glioblastoma; PDAC, pancreatic ductal adenocarcinoma; TAMs, tumor-associated macrophages CAFs, carcinoma-associated fibroblasts.

### Cancer to Non-Cancer

Cancer cells derived exosomes can promote non-cancer cells such as endothelial cells, mesenchymal stem cells (MSCs), carcinoma-associated fibroblasts (CAFs), and immune cells to generate PMNs (86). As a valuable kind of biomolecules, lncRNAs play important roles in various pathophysiological processes of forming PMNs, resulting in malignant tumors initiation and progression.

For example, cancer cells derived exosomes can influence endothelial cells to promote angiogenesis (87) which plays a momentous role in tumor proliferation, and induce vascular permeability (88) which is conducive to tumor metastasis (89). MiR-25-3p, a miRNA transferred from CRC cells to endothelial cells *via* exosomes, promoted vascular permeability and angiogenesis, finally led to hematogenous metastasis in CRC (90). Certainly, exosomal lncRNAs also show significant function in regulating endothelial cells. Bladder cancer (BCa) with lymph node (LN) metastasis has a poor prognosis (91), while PROX1 enhanced lymphatic endothelial cell differentiation and lymphatic budding through constructing interaction with p50 to upregulate VEGFR3 expression level (92). Chen, C. et al. pointed out lncRNA named LNMAT2, which interacted with heterogeneous nuclear ribonucleoprotein A2B1 (hnRNPA2B1) in BCa cells. Under the direction of hnRNPA2B1, LNMAT2 was selectively packed into exosomes and transmitted to human lymphatic endothelial cells (HLECs). Subsequently, LNMAT2 formed a triplex by interacting and integrating with the promoter of PROX1. After epigenetic modification by hnRNPA2B1-mediated H3 lysine 4 trimethylation (H3K4me3), PROX1 transcription was enhanced. Consequently, LNMAT2 mediated lymphangiogenesis and LN metastasis in BCa (67). What’s more, MALAT1 is a well-known lncRNA associated with cancer angiogenesis and metastasis (93). In metastatic epithelial ovarian cancer (EOC), MALAT1 can be transported to human umbilical vein endothelial cells (HUVECs) by exosomes, influencing HUVECs by stimulating the expression of angiogenesis-related genes, such as angiogenin and bFGF (74). Glioma is one of the most malignant cancers of the central nervous system (94). There are numerous literatures relating to exosomal lncRNAs in regulating the angiogenesis of glioma. For example, the long non-coding RNA HOTAIR wrapped by glioma cells’ exosomes induced the proliferation, migration, and tube formation of endothelial cells by increasing the expression of VEGFA (95), a well-known proangiogenic factor (51). Similarly, exosomal lncRNA POU3F3 and CCAT2 also induced angiogenesis in glioma (72, 73).

Furthermore, exosomal lncRNAs delivered to other stromal cells can also change the cells into pro-tumorigenic phenotypes (96). Cancer cells can induce immune tolerance and evade immune surveillance by secreting exosomes (97), which is the major component of PMN creation. For example, γδT cells occupied small proportion of all T lymphocytes but had significant immunosuppressive function as well as positive modulation of immunity (98). Evidence have demonstrated that γδT cell consists an important element of tumor-infiltrating lymphocytes (TILs) and is associated with poor progression and prognosis of breast cancer (99), but considering its positive effect in the innate and adaptive immune systems, a biomarker to identify the truly immunosuppressive subpopulations is urgently requisite. A recent study indicated that exosomal lncRNA SNHG16 was responsible for cross-talk between breast cancer cells and γδ1 T cells, exerting an effect in CD73 expression and resulting in the transformation of γδ1 T cells into the CD73+ immunosuppressive subtype. As a matter of fact, CD73+ γδ1 T cells play a crucial tumor-promoting function in breast cancer microenvironment. As for concrete mechanism, it was speculated that the extraneous SNHG16 activated TGF-β1/SMAD5 pathway by serving as a ceRNA with miR-16-5p in γδ1 T cell (66). Beyond that, macrophages can transform into tumor-associated macrophages (TAMs) under activation by chemokines, inflammatory, and growth factors (100), as well as M1/M2 polarization (101), contributing to the formation of PMNs (102). And evidence have shown exosomal lncRNAs were favorable for this progress. Li, X. et al. figured out that hepatocellular carcinoma (HCC) derived exosomal lncRNA TUC339 induced macrophage activation and polarization. TUC339 was enriched in HCC cells and corresponding exosomes, and over-expression of TUC339 in macrophage cells led to reduced pro-inflammatory cytokine production, decreased co-stimulatory molecule expression, and compromised phagocytosis. Moreover, TUC339 was indispensable for luring M2 polarization. Combined with the transcriptome-wide analysis, cytokine-cytokine receptor interaction et al. may explain the mechanism behind the role of TUC339 (71). LncRNA RUNX2-AS1 was highly expressed in MSCs extracted from multiple myeloma patients (MM-MSCs), and enriched in exosomes of human myeloma cell lines (HMCLs), while RUNX2 was lowly expressed in MM-MSCs. By forming an RNA duplex with RUNX2 pre-mRNA, RUNX2-AS1 interfered RUNX2 pre-mRNA splicing, resulting in the reduction of RUNX2 expression. Ultimately, exosomal lncRNA RUNX2-AS1 mediated decreased osteogenic potential of MSCs, which is the most outstanding character of multiple myeloma (68). In glioma, astrocytes were upon the activation phenotype by exosomes derived from glioma cells carrying lncRNA−ATB, which targeted and suppressed miR-204-3p. And in turn, activated astrocytes promoted the migration and invasion of glioma cells (70).

As noted above, different kinds of cells work together to develop cancer in TME. However, the mutual effects can be bidirectional in some situations. For instance, when HUVECs co-cultured with TAMs derived exosomes, the migration capacity was decreased by targeting miR-146b-5p, which led to the activation of NF-κB phosphorylation. Whereas, when TAMs derived exosomes stimulated HUVECs combining exosomes secreted from EOC cells, the inhibition of HUVECs was reversed. Wu, Q. et al. confirmed two lncRNAs associated with NF-κB phosphorylation in exosomes derived from EOC cells, while the detailed mechanism of exosomal lncRNAs remained unknown (103).

In summary, tumor derived exosomal lncRNAs can transform the state and phenotype of stromal cells to support tumor cells invasion and growth (104). The formation of PMNs triggered by exosomal lncRNAs has become a novel and important focus. Further understanding of their precise mechanisms in stromal cells will provide promising prospects for the prevention and treatment of tumors.

### Cancer to Cancer

Except for connecting the communication between cancer cells and stromal cells, exosomes derived from one cancer cell can also transmitted to another cancer cell under different conditions. For instance, resistant cancer cells can confer drug resistance to sensitive cells through exosomes (77). As a result, the invasion and dissemination of cancers are continually proceeding like virus replication, leading to more malignant phenotypes. Recent studies have indicated that exosomal lncRNAs can contribute to this kind of cell-to-cell communication. For example, Qu, L. et al. demonstrated that lncARSR can disseminate sunitinib resistance in renal cell carcinoma (RCC). Mechanistically, upon the interaction with hnRNPA2B1, lncARSR was specifically sorted into exosomes to drug-sensitive cells. And lncARSR bound miR-34 and miR-449 which targeted to AXL and c-MET as a competing endogenous RNA. The activation of AXL/c-MET caused phosphorylation of AKT, ERK, and STAT3 signal pathway, which counteracted sunitinib’s effect. Therefore, sunitinib-sensitive cells were converted into resistant cells. Furthermore, transcription factors FOXO1 and FOXO3a were phosphorylated and degraded by activated AKT, resulting in increased expression of lncARSR. Intriguingly, the whole process formed a positive feedback loop in RCC cells (47). Long non-coding RNA MRPL23-AS1 was highly expressed in exosomes secreted from salivary adenoid cystic carcinoma (SACC) cells. Besides, the RNA-protein complex consisted of MRPL23-AS1 and EZH2 increased H3K27me3 of the E-cadherin promoter region, causing the initiation of epithelial-mesenchymal transition (EMT). Additionally, exosomal MRPL23-AS1 can also contribute to microvascular permeability in pulmonary microvascular endothelial cells. Altogether, SACC patients tended to lung metastasis and low overall survival upon the role of exosomal MRPL23-AS1 (76). Pancreatic ductal adenocarcinoma (PDAC)is one of the most aggressive cancers (105) because of its low diagnostic rate in the early stage and rapid metastasis (106). Li, Z. et al. elucidated that high invasive PDAC cells can release exosomes carrying lncRNA Sox2ot to low invasive PDAC cells. Then, the internalized Sox2ot promoted EMT and stem cell-like properties by competitively binding the miR-200 family. In addition, Sox2ot embedded in exosomes was validated by orthotopic xenograft assay to confirm that the lncRNA can be used as a special biomarker for PDAC diagnosis and prognosis (78). Accumulated evidence have proofed that hypoxia can remodel primary tumor microenvironment *via* protection from apoptosis (107), activation of EMT (108), abnormal metabolism, as well as microangiogenesis (109), finally, leading to the metastasis of cancer (110). Long non-coding RNA-UCA1 enriched in exosomes derived from hypoxic bladder cancer cells can promote tumor proliferation, migration and invasion though EMT. Regretfully, the authors rarely investigate the detailed function of exosomal lncRNA-UCA1 in bladder cancer cells (79).

As mentioned above, exosomal transmission between cancer cells can increase tumor chemoresistance and metastasis. Since the exosomal lncRNA-mediated intercellular communication occurs in the local area, the study of lncRNA specific antagonists like antisense oligonucleotides (ASOs) (111, 112) will provide powerful aid for conventional chemotherapeutic drugs.

### Non-Cancer to Cancer

In the formation of PMNs, stromal cells like macrophages and fibroblasts were stimulated by “fertilizer” such as inflammatory and immunosuppressive factors, then converted into TAMs and CAFs (113). Reciprocally, the TAMs and CAFs would ulteriorly accelerate PMNs establishment and promote tumor dissemination (114). Exosomes play a significant role in such intercellular communication. Thus, considerable attention has been focused on exosomes in the area of non-cancer cells to cancer cells interflow. And here we emphatically stated the studies about exosomal lncRNAs. HIF-1α-stabilizing long non-coding RNA (HISLA) level was positive correlated with poor overall survival of patients with breast cancer in clinical trial. Chen, F. et al. claimed HISLA wrapped by exosomes derived from TAMs can promote aerobic glycolysis, apoptotic resistance and chemoresistance of breast cancer cells. During this pathophysiological process, HIF-1α, a transcription factor, also played a key role in determining glucose glycolysis or oxidation (115). It is well elucidated that TAMs’ exosomal HISLA competitively bound PHD2, preventing PHD2 having synergistic interaction with HIF-1α. Therefore, the hydroxylation and degradation of HIF-1α were inhibited, leading to enhanced aerobic glycolysis and lactate production. Surprisingly, as the highlight of this study, HISLA in macrophages was upregulated by lactate released from glycolytic cancer cells, which established a feed-forward loop between TAMs and cancer cells (83). It is believed that CAFs play a critical character of matrix remodeling in PMNs formation (116). Previous studies have reported that lncRNA H19 contributed to oncogenesis in many kinds of cancer (117). Moreover, it was found that H19 embedded in exosomes from CAFs enhanced stem cell-like features and chemoresistance in CRC. The RNA-binding protein immunoprecipitation (RIP) experiment and luciferase assay were performed to uncover that H19 sponged miR-141 as ceRNA, resulting in the activation of β-catenin pathway (84). A few lncRNAs that block tumorigenesis were reported as well. Exosome-carrying lncRNA PTENP1 transmitted from normal cells to tumor cells was able to increase cell apoptosis, but decrease motility of BCa cells (85).

In conclusion, stromal cells can deliver lncRNAs to cancer cells *via* exosomes, activating cellular signaling pathways and changing gene expression to accelerate tumor progression. Further studies of this field will offer a novel horizon in exosome associated tumor research, contributing to the delay of tumor deterioration and the improvement of drug efficacy.

## Conclusions And Prospects

**Figure 2:**
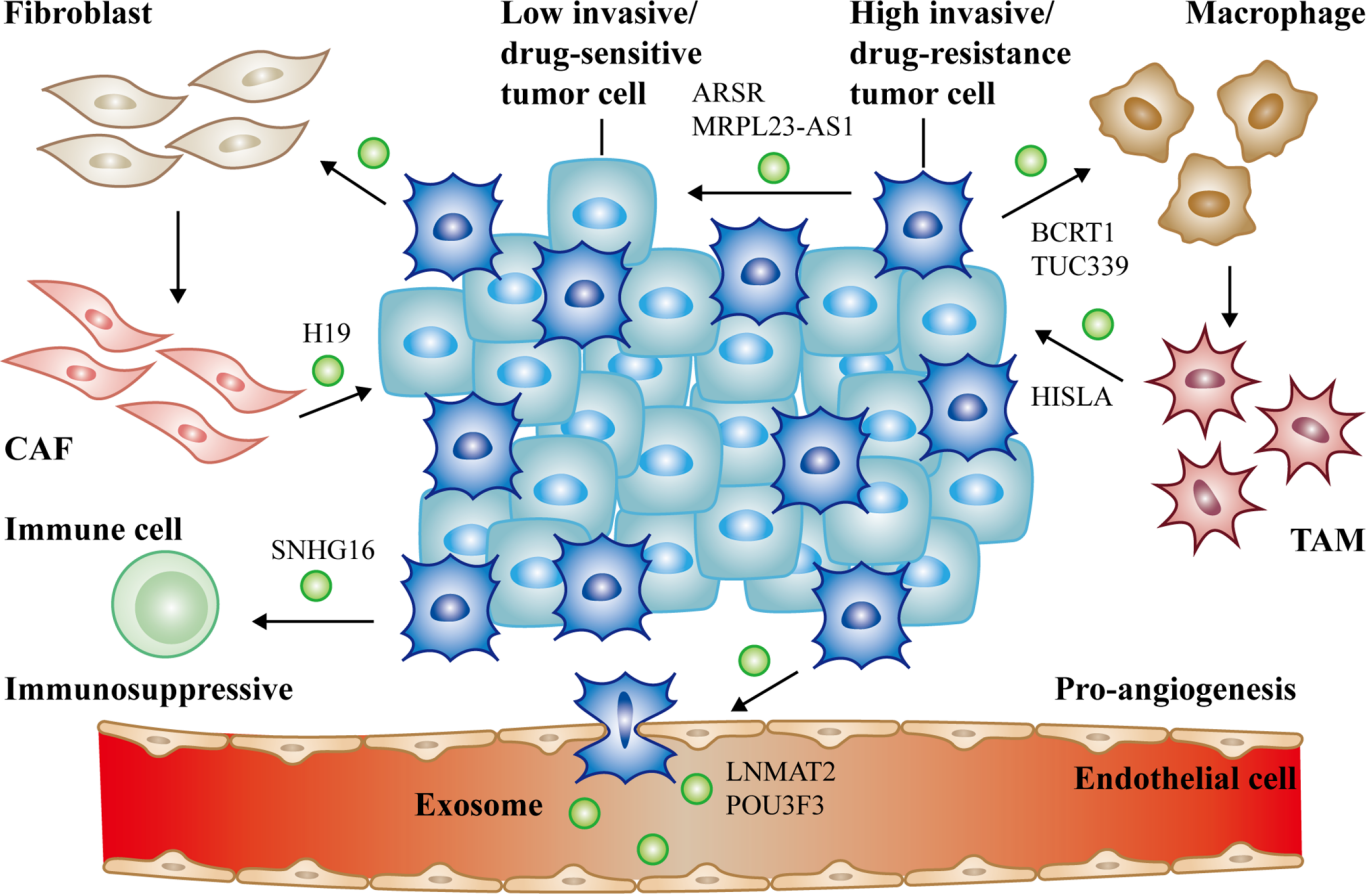
The communication of exosomal long non-coding RNAs (lncRNAs) between cancer cells and non-cancer cells in tumor microenvironment. Cancer cells derived exosomal lncRNAs active fibroblasts, macrophages (lncRNA BCRT1, lncRNA TUC339), endothelial cells (lncRNA LNMAT2, lncRNA POU3F3) and suppress immune cells to form the PMN (lncRNA SNHG16). Reciprocally, activated CAFs (lncRNA H19), TAMs (lncRNA HISLA) can also deliver exosomal lncRNAs to promote cancer progression. Moreover, high/low invasive or drug-resistance/sensitive tumor cells can communicate with each other *via* exosomal lncRNAs as well (lncRNA ARSR, lncRNA MRPL23-AS1). All of the above together promote tumor growth, metastasis, and chemoresistance.

Cancer is the most lethal disease in the world due to poor diagnosis and prognosis. What’s more, it is still not fully clear how cancer grows and colonizes until now. Exosomes represent a new manner of transporting information between cancer cells and other functional cells. lncRNAs play indispensable characters in cancer by regulating gene expression in diverse approaches. Especially, lncRNAs are involved in exosome-mediated intercellular signaling. In this review, we mainly summarized recent literatures about the biological functions and mechanisms of exosomal lncRNAs in tumor microenvironment (**Figure 2**). Meanwhile, we introduced fundamental characteristics and research techniques of exosomes. LncRNA-carrying exosomes from cancer cells or stromal cells can deliver pro-tumorigenesis signals to target cells, contributing to the proliferation, metastasis, angiogenesis and chemoresistance of tumor. Exosomal lncRNAs provide us a novel horizon of tumor generation and development. Since exosomes are non-immunogenic, minimal toxic effects, as well as existed in nearly all of body fluids, they are promising to be applied in clinic such as drug transporters.

However, there are still a number of issues remain poorly understood. For instance, a few studies have revealed that even when lncRNA expression was low in parental cells, it was enriched in exosomes (118, 119). This implicates that lncRNAs are selectively packaged into exosomes through active or passive mechanisms, such as binding to hnRNPA2B1 (47, 67). Nevertheless, the mechanism initially driving lncRNAs to be sorted into exosomes and its relevance with tumor progression are still elusive.

Additionally, a few limitations impede the in-depth exploration of exosomes, and the clinical translation of their functions. First, the results of many studies are only obtained through *in vitro* experiments between the two types of assigned cells and *in vivo* experiments of established animal models (67, 85), which mean that they cannot be confirmed in real pathophysiological conditions. Under these circumstances, if exosomes are served as transport vesicles, the transmission efficiency will not be guaranteed, and the treatment effect may be compromised if exosomes or lncRNAs are used as therapeutic targets. Beyond that, the same lncRNA has been reported to have inconsistent effects in exosomes from different cells and tumors (50, 69, 120). Therefore, it is necessary to control the dose and targeting of exosomal lncRNAs, otherwise, it will affect the homeostasis of the cells and cause great side effects. Based on the above limitations, multiple strategies have been designed for exosome labelling to trace the actual transport path *in vivo*, such as fluorescence (121, 122), bioluminescence (123), and radioactive isotope labelling (124, 125). In addition, the latest studies are focusing on targeted delivery to upgrade the capacity of recipient cells in capturing exosomes, including ligand-receptor binding (126, 127), pH/charge affinity (128, 129), and magnetic attraction (130). As these methods may be genetically altered or inefficient, we need to explore comprehensive techniques for exosome labelling and targeted delivery to maintain specificity, high efficiency, and native function of exosomes. Second, a large proportion of current studies are on the strength of the mechanism and function of cell and animal experiments, coupling with differences in the expression and prognosis of clinical samples (68, 78). Large-sample multicenter and prospective clinical studies are required to evaluate the authenticity and reliability of clinical application. Third, it is difficult to quantify and standardize exosomal lncRNAs in both experimental and clinical applications due to low abundance of lncRNAs in exosomes (131). Thus, a fast and sensitive analytical method is urgently needed.

With more researches conducted on exosome-derived lncRNAs, it is believed that exosomal lncRNAs will not only help us shed more light on the pathophysiology of tumors, but also be widely used in clinical diagnosis and therapeutics in the near future.

## Author Contributions

SH wrote the manuscript and designed the figures. YQ and YL collected the related references and edited the manuscript. XC and HL provided guidance and revised the manuscript. All authors contributed to the article and approved the submitted version.

## Funding

This work is supported by grants from the National Natural Science Foundation of China (No. 81572855 to XC and No. 81202300 to HL) and a project of Hubei Natural Science Foundation of China (No. 2015CFB462).

## Conflict of Interest

The authors declare that the research was conducted in the absence of any commercial or financial relationships that could be construed as a potential conflict of interest.
